# Difference in mobilization of progenitor cells after myocardial infarction in smoking versus non-smoking patients: insights from the BONAMI trial

**DOI:** 10.1186/scrt382

**Published:** 2013-12-24

**Authors:** Guillaume Lamirault, Sophie Susen, Virginie Forest, Caroline Hemont, Angelo Parini, Philippe Le Corvoisier, Christophe Piot, Marie-Jeanne Richard, Béatrice Delasalle, Hélène Rouard, Catherine Sportouch, Virginie Persoons, Eric Van Belle, Jérôme Roncalli, Patricia Lemarchand

**Affiliations:** 1 INSERM UMR1087/CNRS UMR6291, l’institut du thorax, IRS-UN, 8 quai Moncousu, BP 70721 44007 Nantes, cedex 1, France; 2 CNRS, UMR 6291 Nantes, France; 3 Université de Nantes, Nantes, France; 4 CHU Nantes, Laboratoire d’immunologie, CIC 4 Nantes, France; 5 Univ Lille Nord de France, UDSL, IFR 114, EA 2693, Faculté de Médecine, Lille, France; 6 CHRU, Institut d’Hématologie-Transfusion, Pôle de Pathologie cardiologie-vasculaire, Lille, France; 7 INSERM UMR 1048, Inst Metab et Cardiovasc Dis I2MC, Université Toulouse III-Paul Sabatier, Service de Pharmacie CHU Rangueil, Toulouse, France; 8 Inserm, CIC-BT 504, CIC-P 006 and U955 team 3, AP-HP, Henri Mondor University Hospital, Créteil, France; 9 Department of Cardiology, INSERM U661, CHU de Montpellier, Université Montpellier 1, Montpellier, France; 10 Unité Mixte de Thérapie Cellulaire EFS RA, UM biochimie des cancers et Biothérapies, Pôle de Biologie CHU de Grenoble, Grenoble, France; 11 Cell therapy facility EFS Ile de France and CIC-BT N°504, Créteil, France; 12 Department of Cardiology and CIC-Biotherapies 511, CHU de Toulouse, Toulouse, France

## Abstract

**Introduction:**

Although autologous bone marrow cell (BMC) therapy has emerged as a promising treatment for acute myocardial infarction (AMI), trials reported mixed results. In the BONAMI trial, active smoking reduced cardiac function recovery after reperfused AMI. Therefore, we hypothesized that variability in the functionality of BMCs retrieved from patients with cardiovascular risk factors may partly explain these mixed results. We investigated the characteristics of progenitor cells in active smokers and non-smokers with AMI and their potential impact on BMC therapy efficacy.

**Methods:**

Bone marrow and blood samples from 54 smoking and 47 non-smoking patients enrolled in the BONAMI cell therapy trial were analyzed.

**Results:**

The white BMC and CD45dimCD34+ cell numbers were higher in active smokers (*P* = 0.001, *P* = 0.03, respectively). In marked contrast, either bone marrow or blood endothelial progenitor CD45dimCD34 + KDR + cells (EPCs) were decreased in active smokers (*P* = 0.005, *P* = 0.04, respectively). Importantly, a multivariate analysis including cardiovascular risk factors confirmed the association between active smoking and lower EPC number in bone marrow (*P* = 0.04) and blood (*P* = 0.04). Furthermore, baseline circulating EPC count predicted infarct size decrease at three months post-AMI in non-smokers (*P* = 0.01) but not in active smokers. Interestingly, baseline circulating EPCs were no longer predictive of cardiac function improvement in the BMC therapy group.

**Conclusions:**

These data suggest that circulating EPCs play an important role in cardiac repair post-AMI only in non-smokers and that active smoking-associated EPC alterations may participate in the impairment of cardiac function recovery observed in smokers after AMI, an effect that was overridden by BMC therapy.

## Introduction

It has now been more than a decade since clinical scientists began to explore a potential beneficial effect of administering cells to the ischemically injured heart. Stimulated by pioneering experimental studies that showed that bone marrow–derived cells (BMCs) might regenerate infarcted myocardium clinicians quickly translated this concept into clinical applications [1]. Although all clinical trials have reported the remarkable safety of intracoronary administration of autologous BMCs in patients with acute myocardial infarction (AMI), the overall effect on improvement in left ventricle ejection fraction has been modest and significantly less persuasive than the expectations raised by the results obtained in preclinical animal models, reflecting our incomplete understanding of underlying mechanisms [2]. One hypothesis for such discrepancies is wide inter-patient variability in cell functionality. This issue is particularly relevant to the use of autologous bone marrow cells, the function of which is decreased by advanced age [3], and the risk factors for atherosclerosis [4-6] which implies that these patients are at risk of being treated with cells that are intrinsically defective. The prognostic impact of this altered pattern is reflected by the predictive value of the colony forming unit (CFU) capacity of the infused cells on the treatment outcome in chronic ischemic heart disease [7]. Furthermore, in a recent study using transgenic mouse models Wang *et al*. demonstrated that donor myocardial infarction by itself impairs the therapeutic potential of bone marrow cells [8], suggesting that another reason clinical autologous BMC trials have shown conflicting results may be that the BMCs used in the human AMI trials are impaired by the effect of acute cardiac injury on BMCs. In the BONAMI trial [9], we randomized patients with successfully reperfused AMI, residual left ventricular ejection fraction (LVEF) of ≤45% and myocardial viability defects, to intracoronary BMC infusion or state-of-the-art therapy. The primary endpoint of the study was improvement of myocardial viability three months after AMI. We observed a trend in favor of the cell transfer group, with myocardial viability improving in 16/47 patients in the BMC group compared with 7/43 in the control group (*P* = 0.06). Interestingly, a multivariate logistic regression analysis including other major prognostic factors for left ventricular function recovery after AMI also showed that BMCs improve myocardial viability recovery and that active smoking impairs myocardial viability recovery. Thus, we hypothesized that smoking may adversely affect the favorable cell-mediated response.

Therefore, the aim of the current study was to investigate the characteristics of BMCs in smoking and non-smoking patients after AMI in the BONAMI trial and their impact on the efficacy of BMC therapy.

## Methods

### Study design

The study population included 101 patients with AMI recruited from the patient cohort undergoing intracoronary cell infusion (BMC group) or not (control group) from the BONAMI trial (NCT00200707). The ethics review board of the Nantes University Hospital approved both protocols, and studies were conducted in accordance with the Declaration of Helsinki. Written informed consent was obtained from each patient.

Patients admitted with an ST-segment elevation AMI at the University Hospitals of Créteil, Grenoble, Lille, Montpellier, Nantes and Toulouse were enrolled in the BONAMI trial between December 2004 and January 2007 [9]. Enrollment criteria were an age between 18 and 75 years, a successful percutaneous coronary intervention with stent implantation performed on the culprit lesion during the 24 hours after the onset of symptoms, LVEF lower than 45%, absence of myocardial viability in at least 2 contiguous segments out of 17 by 4-hour resting thallium-201-gated-single-photon-emission computed tomography (SPECT), and statin treatment. In addition, baseline echocardiography was performed on days 4 to 7. Patients were randomly assigned in a 1:1 ratio to either the control group (n = 49) or the BMC group receiving cardiac cell therapy (n = 52). A peripheral blood sample was obtained from patients in both groups seven to ten days after AMI. Moreover, a 50 mL bone marrow sample was aspirated the same day from the iliac crest for 51 of the patients in the cardiac cell therapy group. Of this sample, 1 mL was used for biological study and the remainder was used for preparation of the cell therapy product (100 × 10^6^ bone-marrow mononucleated cells). A total of 93/101 peripheral blood samples and 47/51 bone marrow samples were evaluated. All biological samples were transferred at room temperature within 24 hours to Nantes, France, where all biological analyses were performed. Smoking status was evaluated at the time of admission to the hospital (day 0 of AMI). Patients were classified as active smokers, non-smokers (patients who had never smoked) or former smokers (patients who had stopped smoking at least three months before day 0 of AMI).

### Clinical follow-up of patients enrolled in the BONAMI trial

Echocardiography and SPECT were repeated three months after AMI. Two independent core-imaging laboratories blinded to treatment assignment performed all analyses at baseline and at three months. Procedures have been described in detail previously [9].

Three parameters were selected for the present study. First, change in LVEF was measured by echocardiography between baseline and the three-month follow-up. Second, two myocardial viability parameters measured by four-hour resting SPECT were studied: change in infarct size between baseline and the three-month follow-up, and third, myocardial viability improvement which was defined as the number of non-viable segments at baseline that were viable at the three-month follow-up.

### Flow cytometry analysis

Flow cytometric analysis was performed using the BD FACSCalibur flow cytometer (Becton Dickinson, France SAS, Le Pont de Claix, France). Samples of peripheral blood collected in ethylenediaminetetraacetic acid (EDTA) and bone marrow collected in acid-citrate-dextrose (ACD)-containing tubes were analyzed using flow cytometry. Cells were incubated for 20 minutes at room temperature with antibodies against human CD45 (PC7-labeled, Beckman coulter), CD34 (APC-labeled, Beckman coulter, France SAS, Villepinte, France), CD133 (phycoerythrin (PE)-labeled, Miltenyi Biotec, SAS, Paris, France), KDR (PE-labeled, R&D Systems, Abingdon, United Kingdom) and CXCR4 (PE-labeled, Becton Dickinson). Red cells were subsequently lysed with a lysing solution without washing (Facs Lysing Solution™, Becton Dickinson). The inclusion of a calibrated number of Cytocount fluorescent beads (Cytocount beads, Dako, Cytomation SA France, Trappes, France) allowed the absolute positive cell numbers to be enumerated directly from a single flow cytometric measurement.

Analysis was performed with the CellQuest Software (BD Biosciences, San Jose, USA). Quantification of CD45dimCD34+ hematopoietic progenitor cells (HPCs) was performed according to the gating strategy described in the International Society of Hematotherapy and Graft Engineering (ISHAGE) guidelines [See Additional file 1: Figure S1] [10]. Expression of CD133, KDR and CXCR4 markers was studied in CD45dimCD34+ cells. This allows us to distinguish EPC from mature endothelial cells, a cell subpopulation that is CD45- [11]. All analyses were performed by two independent investigators who were blinded to patient groups.

### Preparation of mononuclear cells

Peripheral blood mononuclear cells (PBMCs) were isolated using lymphocyte preparation medium centrifugation procedures (Eurobio). Bone marrow mononuclear cells (BMMCs) were directly obtained from cell therapy product surplus in patients enrolled in the BONAMI trial.

### Colony-forming unit assay for HPCs

PBMCs or BMMCs were seeded in triplicate in methylcellulose-coated plates (Methocult GF H4534; Stem Cell Technologies, Grenoble, France). Plates were studied under phase-contrast microscopy, and granulocyte macrophage colony-forming units (CFU-GM; colonies >40 cells) were counted after 14 days of culture by two independent investigators blinded to patient group.

### Colony-forming unit assay for EPCs

PBMCs or BMMCs were seeded in fibronectin-coated plates (Endocult, Stem Cell Technologies) and cultured at 37°C in 5% CO2. After two days of culture, non-adherent cells were harvested and replated in fibronectin-coated plates. After three days of culture, endothelial cell colony-forming units (CFU-EC) were counted using phase-contrast microscopy by two independent investigators blinded to patient group. Colonies were defined as recommended by the manufacturer as the presence of a central core of round cells surrounded by radiating thin flat cells.

### Migration assay

Granulocyte colony stimulating factor (G-CSF), stromal cell-derived factor (SDF)-1α or vascular endothelial growth factor (VEGF, 100 ng/mL) were added to the culture medium in the lower chambers of 8-micron transwells (Becton Dickinson), and 1 × 10^5^ PBMCs or BMMCs were placed in the upper chambers. After three hours of culture, cells were counted in the lower chamber and the ratio between the number of cells that migrated in the presence or in the absence of G-CSF, SDF-1α or VEGF was calculated. Each experiment was performed in duplicate.

### VEGF quantification

Plasma samples from peripheral blood collected in EDTA-containing tubes were aliquoted and stored at -80°C until use. Plasma VEGF level was measured with an ELISA kit following the manufacturer’s instructions (human VEGF kit; R&D Systems).

### Statistical analysis

All biological results were expressed as mean value with standard deviation. The baseline characteristics and biological data were recorded for each group and compared using t-test or Mann Whitney for continuous variables, and Chi2 test or Fisher’s exact test for categorical variables. Correlations were evaluated by Spearman’s rho or Pearson’s r tests.

A multivariate analysis was performed to identify factors associated with cell numbers (either absolute or relative) using linear regression analysis with a manual selection of factors. Evaluated factors were the following clinical variables: age, sex, major cardiovascular risk factors (hypertension, hyperlipidemia, diabetes, active smoking).

Another multivariate analysis was performed to test if smoking status, EPC numbers and their potential interaction altered cardiac function improvement at three months post-AMI using linear regression analysis with a manual selection of factors. The same multivariate analysis was performed with CD34 + CD45dimCXCR4 or with CFU-EC. Evaluated cardiac function parameters were changes in LVEF, myocardial viability and infarct size measured by SPECT from baseline to three months post-AMI.

*P*-value <0.05 was considered statistically significant. All analyses were performed using SAS 9.2 statistical software.

## Results

### Impact of active smoking on patients with AMI

Baseline characteristics of the patients enrolled in the BONAMI trial grouped according to smoking status are presented in Table 1. Among the 101 randomized patients, 54% were active smokers at the time of AMI. In the former smokers group, time since smoking cessation was 22 ± 13 years, with only one patient having stopped smoking for less than 2 years. As compared to non/former smokers, active smokers were younger with a lower frequency of hypertension, hyperlipidemia and diabetes.

**Table 1:** Baseline characteristics of the BONAMI patients, according to smoking status

**Clinical parameters and risk factors**	**Total (number = 101)**	**Non-smokers and former smokers (number = 47)**	**Active smokers at admission**^**a **^**(number = 54)**	***P*-value**^**b**^
Age (years), mean ± SD	56 ± 11	61 ± 9	51 ± 10	<0.001
Male, n (%)	86 (85)	37 (79)	49 (91)	NS
Body mass index (kg/m^2^), mean ± SD	25.7 ± 3.7	26.3 ± 3.1	25.1 ± 4.1	NS
Hypertension n (%)	35 (35)	23 (49)	12 (22)	0.01
Hyperlipidemia n (%)	41 (41)	25 (53)	16 (30)	0.02
Diabetes n (%)	20 (20)	14 (30)	6 (11)	0.03
Family history of cardiac disease n (%)	39 (39)	22 (47)	17 (31)	NS
Years since smoking cessation (former smokers only), mean ± SD (n)	-	22 ± 13 (23)	-	-
Treatment (Statin), n (%)	101 (100)	47 (100)	54 (100)	-
Mean LVEF, % ± SD	38.9 ± 7.5	39.1 ± 7.7	38.9 ± 7.4	NS

^a^active smoking was evaluated at the time of admission to the hospital (day 0 of AMI). Other patients were non-smokers (patients who had never smoked) or former smokers (patients who had stopped smoking at least three months before study); ^b^Chi-2 test, t-test. LVEF, left ventricular ejection fraction; NS, not significant; SD, standard deviation.

Differences in progenitor cell numbers were analyzed in peripheral blood and bone marrow samples collected 9.3 ± 1.7 days after percutaneous coronary intervention for acute myocardial infarction.

The most important differences in cell subpopulations between active and non/former smokers were observed in the bone marrow (Table 2). First, the white cell (WC) absolute number per microliter was higher in active smokers as compared to non/former smokers (*P* = 0.001). Although the absolute number of hematopoietic progenitor cells (HPCs), including CD45dimCD34+, CD45dimCD34 + CD133+ and CD45dimCD34 + CXCR4+ cells, was increased in active smokers (*P* = 0.02, *P* = 0.005 and *P* = 0.01, respectively), the relative HPC number (expressed as a percentage per 10^5^ WC) was not different between active and non/former smokers. In marked contrast, endothelial progenitor CD45dimCD34 + KDR + cell (EPC) relative number expressed as a percentage per 10^5^ WC was significantly lower in active smokers as compared to non/former smokers (*P* = 0.04). Furthermore, BMCs from active smokers showed a significantly lower migratory response to G-CSF (*P* = 0.03) as compared to BMCs from non/former smoker patients. Finally, measurements of either CFU-GM or CFU-EC were not statistically different between patients with or without active smoking.

**Table 2:** Comparison of biological parameters, according to smoking status

**Cell type**	**Bone marrow**			**Peripheral blood**		
	**Mean ± SD (number)**		***P*-value**^**c**^	**Mean ± SD (number)**		***P*-value**^**c**^
	**Non smokers and former smokers**	**Active smokers at admission**		**Non smokers and former smokers**	**Active smokers at admission**	
**White cells (WC)**						
Leukocytes/μL	10,685 ± 4,818 *(20)*	20,029 ± 9,943 *(27)*	0.001	7,904 ± 2,249 (41)	9,171 ± 2,426 (52)	0.016
**Haematopoietic progenitor cells**						
CD45dimCD34+/μL	49.0 ± 29.8 (20)	83.1 ± 52.2 (28)	0.02	2.7 ± 2.6 (40)	2.8 ± 2.0 (50)	NS (0.9)
CD45dimCD34+/10^5^ WC	399.3 ± 145.4 (20)	419.0 ± 158.9 (27)	NS (0.9)	33.7 ± 26.1 (40)	30.8 ± 20.0 (50)	NS (0.7)
CD45dimCD34 + CD133+/μL	41.8 ± 24.4 (19)	79.6 ± 52.0 (28)	0.005	2.6 ± 2.6 (39)	2.7 ± 1.9 (50)	NS (0.3)
CD45dimCD34 + CD133+/10^5^ WC	394.7 ± 148.8 (19)	402.0 ± 148.5 (27)	NS (0.4)	32.9 ± 26.0 (39)	29.4 ± 19.3 (50)	NS (0.7)
CD45dimCD34 + CXCR4+/μL	43.6 ± 25.7 (19)	82.6 ± 52.9 (28)	0.01	2.6 ± 2.6 (39)	2.7 ± 2.0 (49)	NS (0.5)
CD45dimCD34 + CXCR4+/10^5^ WC	392.1 ± 144.1 (19)	405.5 ± 165.5 (27)	NS (0.9)	32.7 ± 25.6 (39)	29.5 ± 19.7 (49)	NS (0.7)
**Endothelial progenitor cells**						
CD45dimCD34 + KDR+/μL	1.5 ± 0.7 (16)	1.8 ± 1.6 (22)	NS (0.9)	0.2 ± 0.1 (31)	0.1 ± 0.2 (40)	0.02
CD45dimCD34 + KDR+/10^5^ WC	13.5 ± 7.1 (16)	9.1 ± 8.2 (21)	0.04	2.2 ± 1.9 (31)	1.2 ± 1.8 (40)	0.005
**Colony-forming cell assays**						
CFU-GM/1.10^5^ BMMC or /2.10^5^ PBMC	68.5 ± 54.5 (16)	95.7 ± 68.7 (22)	NS (0.2)	13.6 ± 16.6 (39)	18.6 ± 27.8 (53)	NS (0.4)
CFU-EC/1.10^6^ BMMC or /2.10^6^ PBMC	3.9 ± 5.3 (15)	11.6 ± 15.0 (22)	NS (0.1)	3.6 ± 5.7 (34)	2.6 ± 4.4 (50)	NS (0.6)
**Migration assay**						
Spontaneous migration^a^	31,374 ± 8,731 (16)	30,877 ± 8,600 (24)	NS (0.8)	38,613 ± 15,212 (38)	35,383 ± 10,607 (48)	NS (0.4)
G-CSF 100 ng/mL	1.5 ± 0.4 (16)	1.3 ± 0.4 (24)	0.03	1.3 ± 0.4 (38)	1.4 ± 0.3 (48)	NS (0.3)
SDF-1α 100 ng/mL^b^	1.3 ± 0.3 (16)	1.36 ± 0.4 (24)	NS (0.9)	1.28 ± 0.3 (38)	1.31 ± 0.3 (48)	NS (0.6)
VEGF 100 ng/mL^b^	1.32 ± 0.3 (16)	1.49 ± 0.5 (24)	NS (0.3)	1.3 ± 0.3 (38)	1.36 ± 0.4 (48)	NS (0.3)
**VEGF**						
Plasmatic VEGF (pg/mL)	-	-	-	239.5 ± 359.3 (38)	204.7 ± 341.3 (45)	NS (0.6)
**CRP**	-	-	-	23.7 ± 27.5 (26)	28.0 ± 35.7 (39)	NS (0.07)

^a^number of cells that migrated without cytokines; ^b^ratio between the number of cells that migrated in cytokine presence and the spontaneous migration; ^c^Mann–Whitney test. BMMC, bone marrow mononuclear cell; CFU-GM, granulocyte macrophage colony forming units; CRP, C-reactive protein; G-CSF, granulocyte colony stimulating factor; NS, not significant; SD, standard deviation; SDF-1α, stromal cell-derived factor-1α; VEGF, vascular endothelial growth factor.

In peripheral blood, while white blood cell (WBC) count was higher in patients with active smoking (*P* = 0.02, Table 2), there were no significant differences in peripheral blood HPCs according to smoking status. In marked contrast, either absolute or relative number of circulating EPCs were lower in active smokers (*P* = 0.02 and *P* = 0.005, respectively). No correlation was observed between EPC numbers and inflammatory response post-MI, assessed by blood C-reactive protein (CRP) and WBC measurements.

Differences in HPC and EPC number were also analyzed in the presence or absence of major cardiovascular risk factors (age, active smoking, diabetes, hyperlipidemia, hypertension) in peripheral blood and bone marrow. Active smoking was the only factor together with age associated with differences in progenitor cell numbers, in univariate analysis both in bone marrow and in peripheral blood (not shown). Importantly, a multivariate linear regression analysis including cardiovascular risk factors (age, sex, hypertension, hyperlipidemia, diabetes, active smoking) also identified an association between active smoking and a lower relative number of EPCs, in either bone marrow (*P* = 0.04) or blood (*P* = 0.04), demonstrating that the relationship between active smoking and a reduced EPC number was independent of the other cardiovascular risk factors.

Together these data show a greater overall bone marrow activation post-AMI in active smokers. In contrast, there was no increase in EPC number in either bone marrow or blood of active smokers as compared to non/former smokers and no blood mobilization of EPCs, suggesting that there was a lack of specific activation of the endothelial cell lineage in active smokers.

### Correlation between blood EPC number and improvement of cardiac function after AMI

In order to identify potential relationships between blood circulating progenitor cell levels and improvement of cardiac function three months post-AMI, we performed Spearman correlations between baseline blood EPC numbers (absolute and relative) and (1) change in LVEF, (2) change in infarct size and (3) myocardial viability improvement. This analysis was performed separately in patients randomized to BMC therapy (n = 52) and control (n = 49) group, in order to account for a potential independent effect of BMC infusion on cardiac function.

At baseline and three months post-AMI, mean LVEF did not differ between BMC and control groups (38.1 ± 7.9% versus. 39.8 ± 7.0%, at baseline, and 39.1 ± 10.2% versus. 41.5 ± 8.76% at three months post-AMI). Furthermore, mean LVEF was not significantly modified between baseline and three months post-AMI in either BMC or control groups (*P* = 0.14 and *P* = 0.07, respectively).

**Figure 1:**
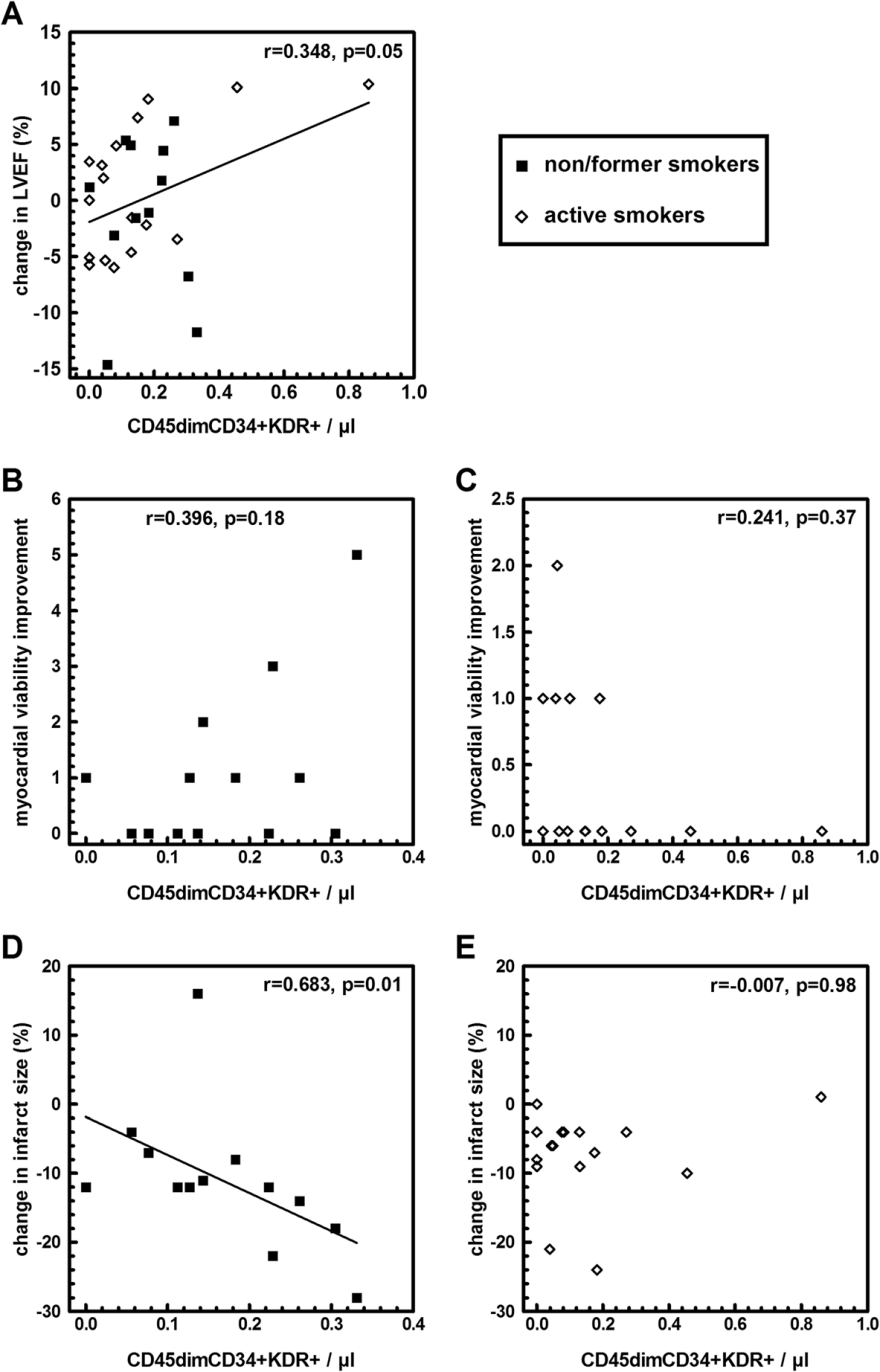
**Correlations between CD45dimCD34 + KDR + cell number at baseline and change in LVEF (A), in myocardial viability improvement (B,C), in infarct size (D,E), in patients of the control group reassessed at three months follow-up.** Black square: former smokers and non-smokers, white rhombus: active smokers at the time of AMI. Myocardial viability improvement was expressed as the number of non-viable segments at baseline that were viable at three months follow-up, measured by four-hour resting SPECT. No significant interaction was observed between cell number, smoking status and LVEF. Correlation between change in LVEF and cell number was significant after adjustment for smoking status (*P* = 0.05). Interaction between CD45dimCD34 + KDR + cell number, smoking status and myocardial viability improvement (measured as the increase in viable segment number) was significant (*P* = 0.02). Interaction between CD45dimCD34 + KDR + cell number, smoking status and infarct size was significant (*P* = 0.01). AMI, acute myocardial infarction; LVEF, left ventricular ejection fraction; SPECT, thallium-201-gated-single-photon-emission computed tomography.

**Figure 2:**
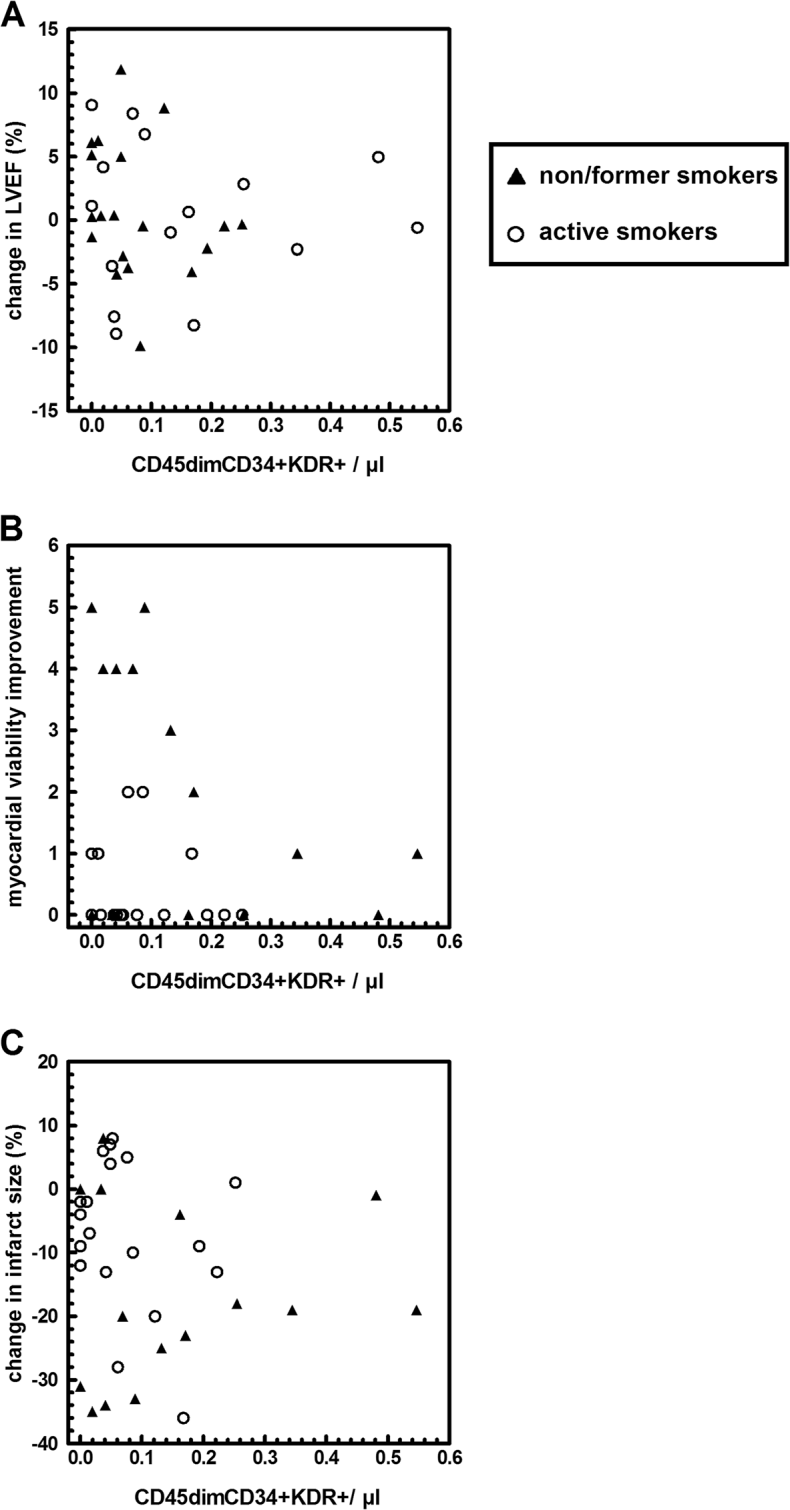
**Correlations between CD45dimCD34 + KDR + cell concentration at baseline and change in LVEF (A), myocardial viability improvement (B) and in infarct size (C) in patients of the cell therapy group reassessed at three months follow-up.** Black triangle: former smokers and non-smokers, white circle: active smokers at the time of AMI. No significant interactions were observed between cell number, smoking status and clinical parameters (change in LVEF: *P* = 0.39, in infarct size: *P* = 0.27, in gain of myocardial viability: *P* = 0.36), nor correlations between cell number and clinical parameters after adjustment for smoking status (LVEF: *P* = 0.68, infarct size: *P* = 0.99, myocardial viability improvement: *P* = 0.06). Myocardial viability improvement was expressed as the number of non-viable segments at baseline that were viable at three months follow-up, measured by four-hour resting SPECT. AMI, acute myocardial infarction; LVEF, left ventricular ejection fraction; SPECT, thallium-201-gated-single-photon-emission computed tomography.

As previously shown [12], change in LVEF at three months post-AMI significantly correlated with the absolute number of circulating EPCs at baseline in the control group (*P* = 0.05, Figure 1A). Importantly, change in LVEF did not correlate with EPC number in the BMC group (Figure 2A).

No such correlation was observed between the number of circulating EPCs and change in infarct size or myocardial viability improvement (data not shown). In addition, no correlation was observed between the number of circulating EPCs, and LVEF, infarct size or myocardial viability at baseline (data not shown). Also, there was no correlation between EPC number and time to reperfusion after AMI, another predictive factor for LVEF improvement after AMI (data not shown).

No correlation was observed between CD34 + CD45dimCXCR4+ cells (absolute numbers and percentage of white cells), in both bone marrow and blood according to tobacco status and cell therapy efficacy, measured by change in LVEF or in myocardial viability from baseline to three months post-AMI (not shown). Similarly, no correlation was observed between CFU-EC, in both bone marrow and blood, according to tobacco status and cell therapy efficacy, measured by change in LVEF or in myocardial viability from baseline to three months post-AMI (not shown).

### Correlation between blood EPC number and myocardial viability after AMI according to smoking status

In order to evaluate the predictive value of smoking status and EPC number on cardiac function improvement at three months post-AMI, we performed a multivariate analysis. In particular, in the BONAMI trial, cardiac function analysis focused on myocardial viability, as this is a reliable parameter for prediction of recovery of cardiac function after revascularization for AMI [13]. This analysis was performed separately in patients randomized to cell therapy (BMC) (n = 52) and the control group (n = 49), in order to account for the potential effect of BMC infusion.

In the control group, there was an interaction between smoking status and the baseline number of circulating EPCs with respect to its relationship to myocardial viability improvement (P = 0.02 and *P* = 0.004 for absolute and relative number, respectively), which was the primary end-point of the BONAMI trial, measured by the gain of viable segments at three months post-AMI on SPECT. However, subgroup analysis by smoking status did not show significant correlation between baseline circulating EPCs and myocardial viability improvement (Figure 1B-C).

Furthermore, in the control group there was also an interaction between smoking status and the baseline number of circulating EPCs with respect to its relationship to the change in infarct size measured by SPECT (*P* = 0.01 and *P* = 0.02 for absolute and relative number, respectively). Subgroup analysis showed that in non/former smokers, a higher absolute number of circulating EPCs was significantly associated with a greater reduction in infarct size measured by SPECT (*P* = 0.01, Figure 1D), whereas this correlation was not found in active smokers (Figure 1E).

Interestingly, in patients from the BMC group, interactions between smoking status and EPC numbers with respect to its relationship to cardiac function improvement (Figure 2B-C) were no longer observed.

## Discussion

Our study shows that patients with active smoking, as compared to non-smoking and former smoking patients, had an increased number of bone marrow cells with an absence of endothelial cell lineage increase after reperfused AMI. Furthermore, the number of circulating EPCs in non-smokers and former smokers was predictive of infarct size decrease measured by SPECT at three months post-AMI, suggesting that circulating EPCs play a significant role after AMI in these patients. In marked contrast, in patients with active smoking, circulating EPCs were less numerous and their migration was impaired. Finally, circulating EPC number was not predictive of myocardial viability improvement at three months post-AMI in this subgroup. These data suggest that active smoking-associated EPC alterations plausibly participate in the impairment of cardiac function recovery observed in smokers after reperfused AMI. Importantly, circulating EPCs were no longer predictive of cardiac function improvement in patients who received BMC therapy, irrespective of their smoking status. This observation suggests that BMC therapy may override the plausible consequences of EPC alterations observed in the active smoking group.

Patient smoking status was suggested as a significant modifier of viability recovery in the BONAMI trial [9]. Therefore, we aimed at studying the impact of patient smoking status on blood and bone marrow content after reperfused AMI. Because EPC are known to be involved in tissue repair after ischemic events and also to be modulated by smoking status, we focused mainly on the role of EPCs on cardiac function recovery after reperfused AMI in both the control and BMC therapy groups. Finally, as compared to our previous publication [9], this new study provides a comparison of both blood and BM cell components in smokers and non-smokers after reperfused AMI, identifies a potential relationship between EPC blood content and cardiac function improvement after AMI in smokers and non-smokers, and analyzes the impact of cardiac cell therapy on this relationship.

### Endothelial progenitor cells and cardiac repair in smokers and non-smoker patients

The number and function of EPCs are regulated by a variety of factors, including cardiovascular risk factors [14]. Among them, smoking critically reduces the number and function of circulating EPCs in chronic coronary disease as well as in active smokers without coronary disease [6]. In our study, the number of either bone marrow or blood CD45dimCD34 + KDR+/10^5^ WC cells were decreased in active smokers and their migratory response impaired, as compared to non/former smoking patients. Circulating EPCs isolated from patients with coronary artery disease have previously been shown to have an impaired migratory response, which is inversely correlated with the number of cardiovascular risk factors [15]. A reduced number of EPCs may be due to a variety of mechanisms, including exhaustion of the pool of progenitor cells in the bone marrow, impaired functional capacity within the bone marrow, reduced mobilization of EPCs, or reduced survival and/or differentiation of mobilized EPCs [6,14]. The presence of ‘good and poor mobilizers’ post-AMI is well-known [16]. Our study is the first to perform a head-to-head comparison of both bone marrow and blood EPCs numbers on active versus non/former smoking patients after AMI, a time point clinically relevant for cardiac repair. Data show reduced numbers of EPCs in blood as well as in bone marrow in active smokers. This supports the hypothesis of bone marrow exhaustion in smokers as compared to non/former smokers. We previously showed in the BONAMI trial that active smoking impaired cardiac function recovery after AMI [9], findings that have also been observed in animal models of tobacco-smoke or nicotine exposure [17,18]. Therefore, we suggest that, in patients not receiving cell therapy, active smoking-related EPC alterations participate in the impairment of cardiac function recovery after acute reperfused ST elevation MI in smokers as compared to non/former smokers. Alternatively, the absence of change in EPC levels associated with myocardial infarction and smoking status could be related to the presence of endothelial dysfunction in chronic smokers [19], as both may be related [20]. Moreover, EPCs from heavy smokers die prematurely during the early phase of culture [21]. Thus, it is likely that active smoking acts on a variety of mechanisms that synergistically culminate in reduced levels of circulating EPCs [12]. We cannot exclude the possibility that the lack of correlation between clinical parameters of cardiac improvement and circulating EPC levels in active smokers may also indicate that levels of circulating EPCs do not reflect endogenous vascular repair capacity in the presence of ongoing smoking–induced endothelial injury.

### Endothelial progenitor cells and cardiac repair after AMI

A large body of evidence has been accumulated suggesting the importance of (1) circulating EPCs, defined as CD45dimCD34 + KDR + cells [11,22,23], in the mechanisms of vascular repair and (2) EPC number as a marker of vascular health and prognosis [14,22]. In this regard, Werner *et al*. demonstrated in 519 patients with coronary artery disease that increased numbers of CD34 + KDR+/10^5^ WBC EPCs were associated with a reduced risk of death from cardiovascular causes and of recurrence of revascularization and hospitalization [24], a result recently confirmed by meta-analysis [25]. By demonstrating a correlation between baseline EPC levels and myocardial viability improvement after AMI in non-smoking patients, our study suggests that EPCs also play a significant role during the specific situation of myocardial repair following AMI.

### Impact of smoking status on the function of EPCs and on cell therapy

BMCs comprise a heterogeneous mixture of cells containing EPCs, HPCs, and other numerous cell types. Our study confirms that AMI not only leads to quantitative but also to qualitative changes in progenitor cells and that mobilization of progenitor cells after AMI is selective for specific cell types [26]. Several authors have recently reported a negative impact of advanced age, risk factors for atherosclerosis and red blood cell contamination on BMC functionality, with important repercussions on their *in vivo* capacity to promote blood flow recovery in a nude mouse model of hind-limb ischemia [16]. Interestingly, in the REPAIR-AMI trial, no association was detected between BMC subpopulation numbers and contractile recovery [27], as in our present study. This might be due to the fact that all of the patients in the BMC group received 10^8^ BMCs, which was identified as the minimum cell number necessary for beneficial effects in a meta-analysis [28]. Thus, to detect a potential direct cell number-related dose–response relationship, it might be necessary to prospectively administer predefined numbers of BMCs varying at least by a factor of 100-fold difference [27]. In our study, the interaction observed between EPC numbers and smoking status with respect to its relationship to cardiac function improvement observed in the control group was absent in the BMC group. This suggests that the relatively high number of injected BMCs through the coronary vasculature, including EPCs [9], may override the potential role of circulating EPC level on cardiac function improvement in the patients receiving BMC therapy, reinforcing the concept of bone marrow exhaustion in the active smoking group. These findings may also suggest that EPCs may not be the most potent active component for cardiac repair in BMCs. Indeed, BMCs is a highly heterogeneous cell therapy product comprising many distinct cell types. Among those, mesenchymal stem cells (MSCs) or c-kit+/lineage- cells also have a potential for cardiac repair after MI. For example, bone-marrow-derived MSCs have been shown to promote cardiac repair by multiple mechanisms. If *in vivo* differentiation of MSCs into cardiomyocytes or vascular cells after cardiac delivery seems to be very limited, MSCs secrete numerous soluble paracrine factors that promote angiogenesis, stimulate resident cardiac progenitor cells for cardiomyogenesis, or inhibit fibrosis or apoptosis (reviewed in [29]). In addition, bone marrow-derived C-kit+/lineage- cells have been shown to activate endogenous cardiomyogenesis through stimulation of endogenous cardiac progenitors [30] and angiogenesis through secretion of soluble paracrine factors [31]. Therefore, future studies are warranted to understand the critical features of bone marrow-derived progenitor cell preparations and of patients with AMI that are predictive of a favorable response to cell transfer [32].

### Effect of AMI and/or smoking cessation on BMC numbers

Surprisingly we observed that active smokers had significant increases in BM leukocyte and HPC numbers as compared to non/former smokers. Importantly, both bone marrow and blood samples were collected nine days after the beginning of AMI, at a time when all patients were staying in a cardiac care unit and had stopped smoking since admission. In an interesting study, Kondo *et al*. showed that circulating EPCs increased at day 7 after smoking cessation and decreased again after resumption of smoking to the level similar to that before cessation [21]. This suggests that BM leukocyte and HPC numbers observed in blood and bone marrow from AMI patients with active smoking were linked to smoking cessation at the time of examination and bone marrow/blood sampling rather than active smoking.

### Potential limitations

We decided to pool non-smokers and former smokers in the same group of patients. Former smokers were defined as patients who had stopped smoking for at least three months at day 0 of AMI. Whereas the impact of past tobacco smoke exposure in former smokers can modify their overall risk of disease for years after smoking cessation, a large body of evidence shows that the risk of coronary events drops rapidly after tobacco smoke exposure cessation. For example, recent data analyzed the impact of smoking ban laws in public places, showing that the incidence of admission for coronary events is reduced within months as compared to immediate pre-law incidence [33-35]. In the population of former smokers in the BONAMI trial, average time since smoking cessation was 22 ± 13 years. Furthermore, all but one patient had stopped smoking for two or more years at the time of admission for AMI.

Our study did not include viability and apoptosis markers in the EPC flow quantification, both important variables that can modulate efficacy of cardiac cell therapy [36]. As of now there is no standardized protocol for multicenter evaluation of apoptosis on EPCs. Therefore, apoptosis quantification was not performed in the cell therapy product for all patients as a part of its qualification and validation. The number of BM CFU-EC did not correlate with cell therapy efficacy (evaluated by change in LVEF or myocardial viability), a result which is not in agreement with previous studies demonstrating a correlation between CFU-EC and neovascularization potential [37] or ulcer healing [38]. Since in our study we only quantified CFU-EC number, we cannot exclude a functional impairment of these cells according to tobacco status or cell therapy efficacy as it has previously been shown in coronary artery disease [39] or in acute myocardial infarction [8].

Our study is observational and indeed no hypothesis was experimentally tested. One option to gain further insight into the impact of active smoking on EPCs and cardiac repair after AMI would be to use animal models. However, current models for tobacco smoke exposure do not replicate active smoking in humans. Animals are acutely exposed to tobacco smoke for a few hours every day for several weeks, whereas typical active smokers with AMI have been smoking for years or decades. Cigarette consumption is also more broadly distributed during the day as compared to a typical three-hour exposure for animal models. Therefore, findings from these animal studies would potentially be misleading [40].

## Conclusions

Our study emphasizes the potential role of EPCs in the process of cardiac repair after AMI and shows that active smoking impairs both bone marrow and EPC numbers and functionality with plausible deleterious consequences on cardiac function recovery after AMI. Interestingly, BMC intracoronary infusion seems to override the consequences of these alterations. Together, our data suggest that these active smoking-related EPC alterations may be a consequence, at least in part, of bone marrow exhaustion.

## Abbreviations

ACD: acid-citrate-dextrose; AMI: acute myocardial infarction; APC: allophycocyanin; BMC: bone marrow-derived cell; BMMC: bone marrow mononuclear cell; BONAMI: Bone marrow in acute myocardial infarction; CFU: colony forming unit; CFU-EC: endothelial cell colony-forming unit; CFU-GM: granulocyte macrophage colony-forming unit; CRP: C-reactive protein; CXCR4: C-X-C chemokine receptor type 4; EDTA: ethylenediaminetetraacetic acid; ELISA: enzyme-linked immunosorbent assay; EPC: endothelial progenitor CD45dimCD34 + KDR + cell; G-CSF: granulocyte colony-stimulating factor; HPC: hematopoietic progenitor cell; ISHAGE: International Society of Hematotherapy and Graft Engineering; KDR: kinase insert domain receptor; LVEF: left ventricular ejection fraction; MSC: mesenchymal stem cell; PBMC: peripheral blood mononuclear cell; PC7: phycoerythrin-cyanine7 conjugate; PE: phycoerythrin; SPECT: thallium-201-gated-single-photon-emission computed tomography; VEGF: vascular endothelial growth factor; WBC: white blood cell; WC: white cell.

## Competing interests

The authors declare that they have no competing interests.

## Authors’ contributions

GL contributed to the study design, clinical follow-up, data analysis and writing of the report. SS contributed to the study design, data analysis, and writing of the report. VF contributed to the study design, FACS analysis, data analysis and writing of the report. CH contributed to the study design, FACS analysis, data analysis and writing of the report. AP contributed to the study design and data analysis. PLC contributed to patient enrollment and clinical follow-up and writing of the report. CP contributed to patient enrollment and clinical follow-up. MJR contributed to cell product preparation. BD contributed to statistical analysis. HR contributed to cell product preparation and data analysis. CS contributed to patient enrollment and clinical follow-up. VP contributed to cell product preparation and data analysis. EVB contributed to patient enrollment, clinical follow-up, data analysis and writing of the report. JR contributed to patient enrollment, clinical follow-up, data analysis and writing of the report. PL contributed to the study design, data analysis and writing of the report. All authors read and approved the final manuscript.

## Supplementary Material

Additional file 1 **Analysis of hematopoietic and endothelial progenitors cells in peripheral blood by flow cytometry, (A) selection of 5 × 10**^**5**^** CD45+ on the basis of CD45 expression and side scatter optic properties, (B) gating of the CD34+ cells on the basis of CD34 expression and side scatter optic properties, (C) CD34+ absolute number quantification using gating of CytoCount beads, (D) and (E) gating of the CD34 + CD133+ and CD34 + CXCR4+ hematopoietic progenitor cells (HPCs), respectively, (F) gating of the CD34 + KDR + endothelial cells (EPCs).** Click here for file
