# Supplementary material for: Difference in mobilization of progenitor cells after myocardial infarction in smoking versus non-smoking patients: insights from the BONAMI trial
Source: Stem Cell Res Ther. 2013 Dec 24;4(6):152. doi: 10.1186/scrt382 (PMC4054959; doi:10.1186/scrt382)
Supplement: Additional file 1 — Analysis of hematopoietic and endothelial progenitors cells in peripheral blood by flow cytometry, (A) selection of 5 × 10 5 CD45+ on the basis of CD45 expression and side scatter optic properties, (B) gating of the CD34+ cells on the basis of CD34 expression and side scatter optic properties, (C) CD34+ absolute number quantification using gating of CytoCount beads, (D) and (E) gating of the CD34 + CD133+ and CD34 + CXCR4+ hematopoietic progenitor cells (HPCs), respectively, (F) gating of the CD34 + KDR + endothelial cells (EPCs). [file scrt382-S1.doc]

Analysis of hematopoietic and endothelial progenitors cells in peripheral blood by flow cytometry


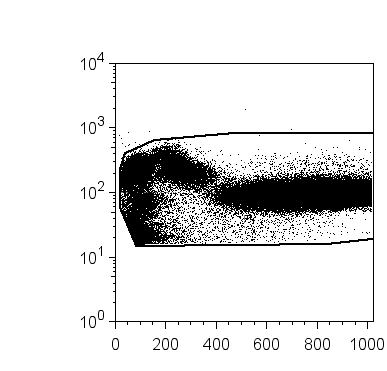

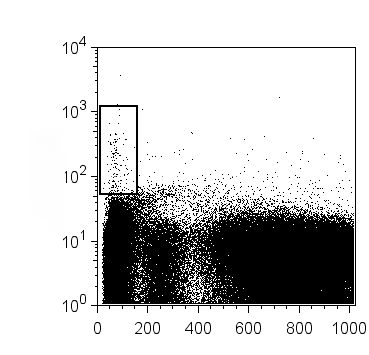

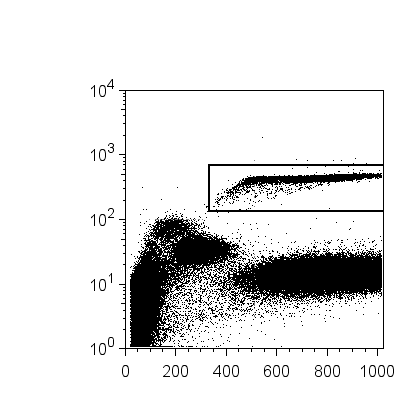

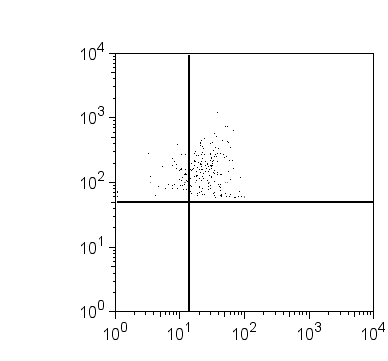

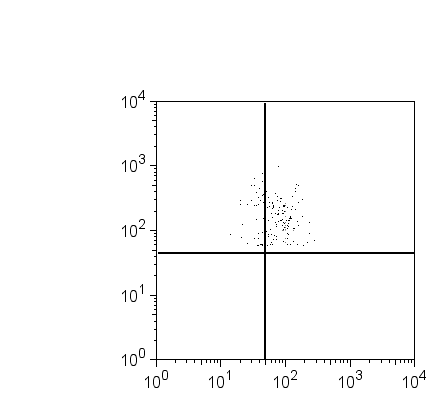

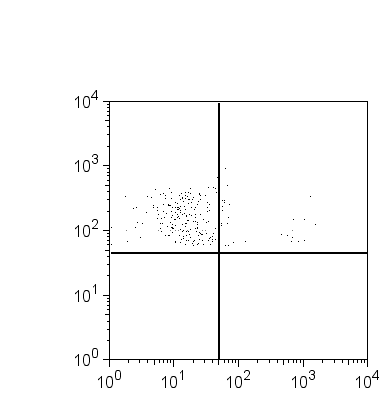


**A**

**B**

**C**

**D**

**E**

**F**

**CD45**

**SSC Height**

**SSC Height**

**SSC Height**

**CD34**

**FL1 H**

**CD34**

**CD34**

**CD34**

**CD133**

**CXCR4**

**KDR**
